# Genomic Prediction Accuracies for Growth and Carcass Traits in a Brangus Heifer Population

**DOI:** 10.3390/ani13071272

**Published:** 2023-04-06

**Authors:** Sunday O. Peters, Kadir Kızılkaya, Mahmut Sinecen, Burcu Mestav, Aranganoor K. Thiruvenkadan, Milton G. Thomas

**Affiliations:** 1 Department of Animal Science, Berry College, Mount Berry, GA 30149, USA; 2 Department of Animal Science, Faculty of Agriculture, Aydin Adnan Menderes University, Aydin 09100, Turkey; 3 Department of Computer Engineering, Faculty of Engineering, Aydin Adnan Menderes University, Aydin 09100, Turkey; 4 Department of Statistics, Faculty of Arts and Sciences, Çanakkale Onsekiz Mart University, Terzioğlu Campus, Çanakkale 17100, Turkey; 5 Department of Animal Genetics and Breeding, Veterinary College and Research Institute, Tamil Nadu Veterinary and Animal Sciences University, Salem 637002, Tamil Nadu, India; 6 Texas A&M AgriLife Research, Beeville, TX 78102, USA

**Keywords:** accuracy, GBLUP, Bayesian methods, genomic prediction, k-means clustering, growth and carcass traits

## Abstract

**Simple Summary:**

The genomic estimated breeding value (GEBV) using data from Brangus heifers were obtained from genomic selection (GS) methods associating the single nucleotide polymorphisms (SNP) marker genotypes with phenotypic data for economically important growth (birth, weaning, and yearling weights) and carcass (depth of rib fat, and percent intramuscular fat and longissimus muscle area) traits using the linkage disequilibrium (LD) between SNP markers and quantitative trait loci (QTL) and/or the genomic relationship between animals. The heritability estimates were found similar across genomic best linear unbiased prediction (the GBLUP), and the Bayesian (BayesA, BayesB, BayesC and Lasso) GS methods for k-means and random cluster. The Bayesian methods resulted in underestimates of heritabilities and overestimates of accuracy of GEBV. However, the GBLUP method resulted in more reasonable estimates of heritabilities and accuracies of GEBV for growth and carcass traits of heifers from a composite population.

**Abstract:**

The predictive abilities and accuracies of genomic best linear unbiased prediction (GBLUP) and the Bayesian (BayesA, BayesB, BayesC and Lasso) genomic selection (GS) methods for economically important growth (birth, weaning, and yearling weights) and carcass (depth of rib fat, apercent intramuscular fat and longissimus muscle area) traits were characterized by estimating the linkage disequilibrium (LD) structure in Brangus heifers using single nucleotide polymorphisms (SNP) markers. Sharp declines in LD were observed as distance among SNP markers increased. The application of the GBLUP and the Bayesian methods to obtain the GEBV for growth and carcass traits within k-means and random clusters showed that k-means and random clustering had quite similar heritability estimates, but the Bayesian methods resulted in the lower estimates of heritability between 0.06 and 0.21 for growth and carcass traits compared with those between 0.21 and 0.35 from the GBLUP methodologies. Although the prediction ability of the GBLUP and the Bayesian methods were quite similar for growth and carcass traits, the Bayesian methods overestimated the accuracies of GEBV because of the lower estimates of heritability of growth and carcass traits. However, GBLUP resulted in accuracy of GEBV for growth and carcass traits that parallels previous reports.

## 1. Introduction

The availability of high-density SNP genotypes from high-throughput genotyping technologies [1,2,3,4] and the development of linear and nonlinear methods (such as the GBLUP, BayesA, BayesB, BayesC, and Bayesian Lasso) [1,5,6] have made genomic selection applicable for the economically important traits in animal and plant breeding [7,8,9,10,11,12,13,14]. Genomic selection methods associate SNP marker genotypes with phenotypic data for economically important traits to obtain the GEBV of animals based on the LD between SNP and QTL and/or genomic relationship among animals. The accuracy of GEBV, important for the genetic progress in GS, is influenced by many factors, including the level of LD between SNP and QTL, heritability of the trait, and the estimation methods of GEBV [15,16,17]. Habier et al. [18] reported that the accuracies of GEBV depend on LD among SNP and QTL, and on genomic relationships among animals in the training and validation datasets. Their findings indicated that the accuracy of GEBV of a selected animal decreased as the genomic relationship between selection animals (candidates) and training animals decreased. Saatchi et al. [19] also showed that if the genetic relationships between animals in training and animals in validation data were minimized as per the pedigree-base additive genetic relationships among animals in the k-means clustering procedure, accuracies of GEBV of animals in the validation data were less affected by their genomic relationships. Villumsen et al. [20] also studied the effect of heritability on the accuracy of GEBV in GS using simulated data and reported that the accuracy of GEBV increased about 17% as the heritability increased from 0.02 to 0.30 in the GS study. Clark et al. [21] compared the accuracy of GEBV from BLUP, the GBLUP, and the BayesB methods, finding that the accuracies of genomic prediction from GS methods depended on the significant effect of QTL on the trait, and that the small effect of QTL resulted in a non-significant difference between GBLUP and BayesB.

The objectives of this research were to characterize LD structure of Brangus heifers and to compare the predictive ability and accuracy of the GBLUP and the Bayesian methods for economically important growth (birth, weaning, and yearling weights) and carcass (depth of rib fat, percent of intramuscular fat, and longissimus muscle area) traits using BovineSNP50 Infinium BeadChip SNP markers (*n* = 54,001 SNP).

## 2. Materials and Methods

### 2.1. Phenotypes

Birth weight (BW), weaning weight (WW), and yearling weight (YW) were phenotypes for growth traits, and depth of rib fat (FAT), percent intramuscular fat (IMF), and longissimus muscle area (LMA) were phenotypes for carcass traits from yearling ultrasound evaluation. Phenotypes were collected from 738 Brangus heifers that were registered with International Brangus Breeders Association [9,22,23]. Year of birth (2005 to 2007), season of calving (spring or autumn), and age of dam were also obtained from the database of the International Brangus Breeders Association. The descriptive statistics of these growth and carcass traits are presented in Table 1.

### 2.2. SNP Marker Genotypes

BovineSNP50 Infinium BeadChips for 54,001 SNP markers were used to genotype each heifer [2]. Genotypes of SNP markers were determined in the A/B allele format and coded as 0, 1, or 2, based on the number of B alleles at each locus. With this SNP marker information and using the snpReady package in R-program [24], three filters were applied for quality control in the following sequence: (a) Animals with > 50% missing data were removed; (b) SNP markers with > 5% missing data or < 95% call rate were removed; and (c) SNP markers with < 10% minor allele frequency were removed. After executing imputation for missing SNP markers, the complete SNP genotype data included 35,351 SNP markers from 738 animals. On each chromosome, the distribution of the number of SNP markers within a 1 Mb window was determined by using the rMVP package in the R-program [25].

### 2.3. Linkage Disequilibrium

The success of GS and genome-wide association studies (GWAS) are dependent on LD, which is a non-random association among SNP markers. LD is measured using the square of correlation ($r^{2}$) between SNP markers and ranges between 0 and 1. Linkage disequilibrium is expressed as

(1) $$r_{ij}^{2}=\frac{{\left(p_{AB}-p_{A}p_{B}\right)}^{2}}{\left(p_{A}p_{a}\right)\left(p_{B}p_{b}\right)}$$

where $p_{AB}$, $p_{A}=1-p_{a}$ and $p_{B}=1-p_{b}$ are the observed frequencies for haplotype AB and alleles A and B at locus i and j, respectively. The estimates of LD for pairwise combinations of all SNP markers were obtained from the *pairwise LD* function of the Synbreed package in the R program [26,27].

### 2.4. Genomic Selection

#### 2.4.1. Genomic Best Linear Unbiased Prediction (GBLUP)

The model for GEBV was:

(2) y=Xb+Zg+e

where y was a vector of BW, WW, YW, LMA, IMF, or FAT; X was a design matrix allocating BW, WW, YW, LMA, IMF or FAT to the fixed effects of overall mean, contemporary groups and dam age; Z was a design matrix allocating BW, WW, YW, LMA, IMF, or FAT to additive genetic effects of animals; b was a vector of fixed effects of overall mean, contemporary groups, and dam age; and g was a vector of additive genomic breeding values for animals following a multivariate normal distribution $g\sim N\left(0,\;G\sigma_{g}^{2}\right)$ with genomic relationship matrix (G) and the additive genetic variance ($\sigma_{g}^{2}$) among animals. e was a vector of residuals following a multivariate normal distribution $e\sim N\left(0,\;I\sigma_{e}^{2}\right)$ with the residual variance ($\sigma_{e}^{2}$).

The G matrix indicating the realized relatedness among animals was calculated as

(3) $$G=\frac{{WW}^{T}}{2\sum_{i=1}^{k}p_{i}\left(1-p_{i}\right)}$$

where W=M−P, M was the (n×k) matrix of SNP markers for the n=738 animals with the k=35,351 SNP markers; P was the (n×k) matrix of the allele frequencies multiplied by 2; $p_{i}$ was the allele frequency of SNP marker i; and the sum was, overall, loci [18,28].

The GBLUP used for the GEBV of animals was equivalent to solving the mixed model equations:

(4) $$\left[\begin{array}{cc} X^{T}X & X^{T}Z\\ Z^{T}X & Z^{T}Z+G^{-1}\frac{\sigma_{e}^{2}}{\sigma_{g}^{2}} \end{array}\right]\left[\begin{array}{c} b\\ g \end{array}\right]=\left[\begin{array}{c} X^{T}y\\ Z^{T}y \end{array}\right]$$

where $\sigma_{g}^{2}$ and $\sigma_{e}^{2}$ were the additive genetic and residual variances and $G^{-1}$ was the inverse of the G matrix. Therefore, the heritability of the trait was defined as $h^{2}=\sigma_{g}^{2}/\left(\sigma_{g}^{2}+\sigma_{e}^{2}\right)$.

The BGLR package (https://cran.r-project.org/web/packages/BGLR/index.html (accessed on 10 March 2022)) in the R program [6,26] was used to solve the mixed model equations in Equation (4) for b and g by estimating the additive genetic and residual variances (${\hat{\sigma }}_{g}^{2}$ and ${\hat{\sigma }}_{e}^{2}$). The estimate of heritability was then calculated as ${\hat{h}}^{2}={\hat{\sigma }}_{g}^{2}/\left({\hat{\sigma }}_{g}^{2}+{\hat{\sigma }}_{e}^{2}\right)$.

#### 2.4.2. The Bayesian BayesA, BayesB, BayesC and Lasso Methods

The Bayesian (BayesA, BayesB, BayesC, and Lasso) methods were applied to estimate the SNP effects for genomic prediction using cross-validation datasets of BW, WW, YW, LMA, IMF, and FAT. The cross-validation data of BW, WW, YW, LMA, IMF, and FAT were modeled as a function of the individual SNP effects:

(5) y=Xb+Mm+e

where y was a vector of BW, WW, YW, LMA, IMF, or FAT; X was a design matrix allocating BW, WW, YW, LMA, IMF, or FAT to the corresponding fixed effects of overall mean, contemporary groups, and dam age; M was a n×k matrix of SNP (0, 1 or 2); b was a vector of fixed effects of overall mean and contemporary groups and dam age; and m was a k×1 vector of SNP effects assumed a priori to follow a multivariate normal distribution m~N(0, Ω) with $\Omega =diag\left(\sigma_{m_{1}}^{2},\sigma_{m_{2}}^{2},\cdots ,\sigma_{m_{k}}^{2}\right)$ the diagonal matrix and $\sigma_{m_{i}}^{2}$ the variance of SNP i. The prior distribution of SNP effect $m_{i}$ depended on the SNP variance $\sigma_{m_{i}}^{2}$ and the prior probability π that SNP i had zero effect:

(6) $$m_{i}|\pi ,\sigma_{m_{i}}^{2}\{\begin{array}{ll} =0 & with\;probability\;\pi \\ \sim N\left(0,\sigma_{m_{i}}^{2}\right) & with\;probability\;\left(1-\pi \right). \end{array}$$

where the parameter of π was defined between 0 and 1 [5]. The specifications for π and the SNP variance $\sigma_{m_{i}}^{2}$ determined the methods of BayesA, BayesB, and BayesC. In BayesA and the BayesB methods, the SNP variance $\sigma_{m_{i}}^{2}$ denoted the ith SNP variance, which had a scaled inverse chi-square distribution ($\chi^{-2}\left(\nu ,S\right)$) with degrees of freedom ν and scale S parameters. These specifications result in a univariate Student’s t distribution t(0,ν,S) for the marginal distribution of the SNP effect $m_{i}|\;\nu ,S$ with the probability of the parameter of (1−π) [5,6]. In BayesC, with the SNP variance $\sigma_{m_{i}}^{2}=\sigma_{m}^{2}$, prior distributions of the SNP effects had a common variance distributed with $\chi^{-2}\left(\nu ,S\right)$. Therefore, these specifications resulted in a mixture of multivariate Student’s t distributions t(0,ν,IS) for the marginal distribution of the SNP effect $m_{i}|\;\nu ,S$ with the probability parameter of (1−π) [5,6]. In the BayesA method, the value of zero was assigned for the parameter of π, resulting in all k SNP in the model. However, in the BayesB and the BayesC methods, the fixed value of 0.95 was assigned for the parameter of π, resulting in 5% of k SNP markers with none-null variances in the model. In the Bayesian Lasso (BL), all k SNP (π=0) were in the model, as in the BayesA method, and each SNP marker variance $\sigma_{m_{i}}^{2}$ had a Laplace distribution $Exp\left(\frac{\lambda^{2}}{2}\right)$ with λ parameter, which had a conjugate prior distribution of Gamma. These specifications result in a Double Exponential (DE) distribution for the marginal distribution of SNP effect $m_{i}|\;\lambda^{2}$ with the probability the parameter of (1−π) [6,29]. The vector of e represented normally distributed residuals ($e\sim N\left(0,\;I\sigma_{e}^{2}\right)$) with the variance ($\sigma_{e}^{2}$), which has a $\chi^{-2}\left(\nu_{e},S_{e}\right)$ with degrees of freedom $\nu_{e}$ and scale $S_{e}$ parameters. The BGLR package (https://cran.r-project.org/web/packages/BGLR/index.html (accessed on 10 March 2022)) in the R program [6,26] was used to estimate SNP effects for BW, WW, YW, LMA, IMF, and FAT.

#### 2.4.3. K-Means and Random Clustering

The animals for cross-validation were divided into 10-fold data sets by using the k-means clustering approach. K-means clustering maximizes genetic relatedness within each cross-validation set and minimizes it between cross-validation datasets based on the genetic dissimilarity (D) matrix among animals [19], which was calculated from the pedigree numerator relationship (A) matrix [30]:

(7) $$d_{ij}=1-\frac{a_{ij}}{\sqrt{a_{ii}a_{jj}}}=1-r_{ij}\;$$

where $d_{ij}$ was a measure of genetic dissimilarity between individuals i and j, $a_{ij}$ was the additive genetic relationship between individual i, and j, $a_{ii}$ ($a_{jj}$) was the $i^{th}$ ($j^{th}$) diagonal element of the A matrix, which represented Wright’s coefficient of relationship ($r_{ij}$) between individuals i and j. *GeneticsPed* package in the R-program [31] was used to create the pedigree numerator relationship (A) matrix, and the *factoextra* package in the R-program [32] implementing the Hartigan and Wong [33] algorithm was used for k-means clustering.

For random clustering, the animals were randomly divided into 10-fold datasets for cross-validation, and this procedure was replicated five times.

#### 2.4.4. Accuracy of Genomic Prediction

The training process in the 10-fold cross-validation from k-means and random clustering approaches was performed by excluding one validation set to train on the remaining nine validation sets, and then the GEBV of animas in the omitted validation set were obtained. The predictive ability of the GBLUP and the Bayesian BayesA, BayesB, BayesC, and Lasso methods in the 10-fold datasets for cross-validation were determined using Pearson’s correlation coefficient ($r_{y,\hat{y}}$) between the observed (y) and predicted ($\hat{y}$) phenotypic values for BW, WW, YW, LMA, IMF, and FAT.

The accuracy of GEBV represented the correlation ($r_{BV,GEBV}$) between the breeding values (BV) and GEBV. However, the BV of animals are unknown, and the accuracy of GEBV of animals for traits was calculated by pooling estimates from the 10-fold cross-validation strategy. The accuracy of the GEBV of animals for traits was estimated using Pearson’s correlation coefficient ($r_{y,\hat{y}}$) weighted by the heritability ($h^{2}$) of the traits in the validation datasets [34]:

(8) $$r_{BV,GEBV}=\frac{r_{y,\hat{y}}}{\sqrt{h^{2}}}$$

## 3. Results and Discussion

### 3.1. Distribution of SNP Markers and LD Analysis

**Figure 1:**
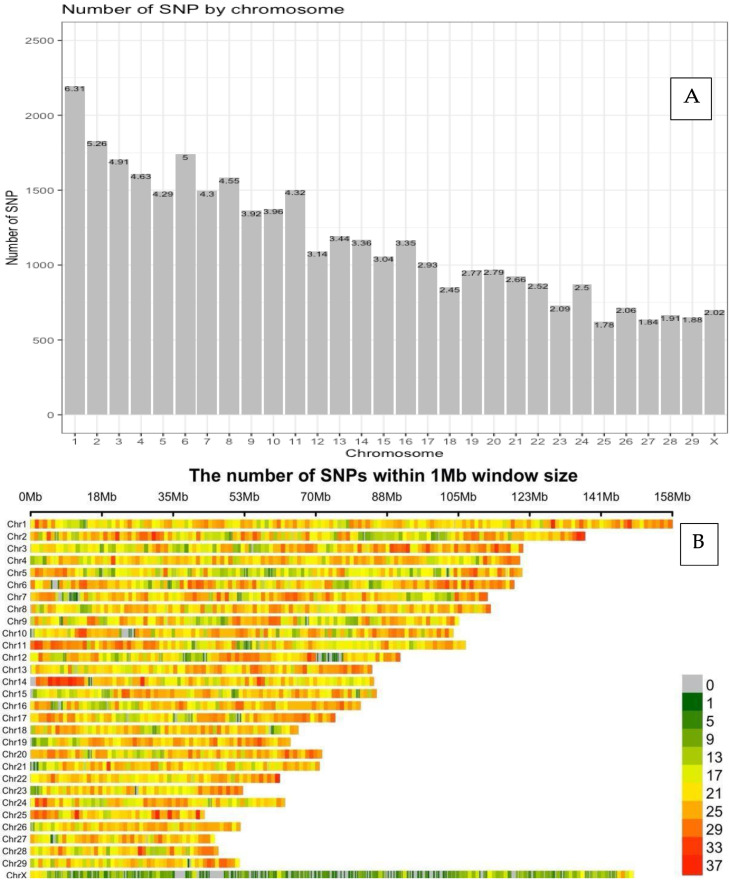
Distribution and density plot of SNP markers on each chromosome. (**A**): Number of SNP markers on each chromosome. (**B**): Density of SNP markers on each chromosome.

We retained 35,351 SNP after filtering markers based on the quality-control criteria. The distribution and density plots of SNP markers per chromosome are presented in Figure 1A,B. The total length of the autosomal genome was 2509.0 Mb, with the shortest chromosome (i.e., 25) being 42.9 Mb in length and the longest chromosome (i.e., 1) being 158.2 Mb in length. The length of chromosome X was 148.6 Mb. As seen in Figure 1A, there was a decreasing trend in the number of SNP markers from chromosome 1 to chromosome X and the SNP coverage ranged between 620 (1.78%) on chromosome 25 and 2194 (6.31%) on chromosome 1. Chromosome 1 and 25 had the longest and the shortest chromosomes with 158.49 Mb and 42.91 Mb in a study of Sahiwal cattle [35] with 157.78 Mb and 42.21 Mb in Charolais, Limousine, and Blonde d’Aquitaine cattle [36], and with 158.03 Mb and 42.80 Mb in Vrindavani crossbred cattle in India [37]. Singh et al. [37] also reported that since the distribution of SNP was related with the length of chromosomes, chromosome 1 had the highest number of SNP (2798) and chromosome 25 had the least number of SNP (792). The largest distance between SNP markers was 3.26 Mb on chromosome 10, and the shortest distance was 0.01 kb on chromosome 15. The average distance between SNP markers was 57.24 kb. Lu et al. [38] reported that the total genome length for Angus, Charolais, and Crossbred beef cattle in Canada was between 2534.98 and 2535.30 Mb, with the shortest chromosome 25 being 42.72 Mb and the longest chromosome 1 being 158.09 Mb. The distribution of the number of SNP differed from 2026 to 2176 for the chromosome 1 and from 580 to 607 for chromosome 28, and the overall average distance between two adjacent SNP markers was 70 kb.

The density plot of SNP markers in Figure 1B showed the number of SNP markers within a 1 Mb window on each chromosome. The horizontal axis of the density plot of SNP markers indicates the length of chromosome (Mb). The different color shows SNP density from 0 to 37 SNP markers on each chromosome. The distribution of SNP markers on the autosomal chromosomes was not uniform and indicated a tendency of being clustered in some regions. The colors on the chromosomes showed the variation in the density of SNP markers on each chromosome. The density of the SNP markers differed from 12.0 SNP/Mb on chromosome 12 to 15.1 SNP/Mb on chromosome 19. For the X chromosome, density of the SNP markers was 4.7 SNP/Mb. Chromosome 1 had a similar density pattern of SNP; however, chromosomes 11, 14, 24, and 25 had higher density of SNP at the beginning of the chromosomes compared to the rest of the chromosomes. The X chromosome was the second largest chromosome, but green and grey colors indicated very sparse densities of SNP markers. In addition, chromosome 6 had more SNP markers than chromosomes 3, 4, and 5, and was shorter than those chromosomes; therefore, the density of SNP markers on chromosome 6 (14.6 SNP/Mb) was higher than those on chromosomes 3 (14.1 SNP/Mb), 4 (13.3 SNP/Mb) and 5 (112.3 SNP/Mb).

**Figure 2:**
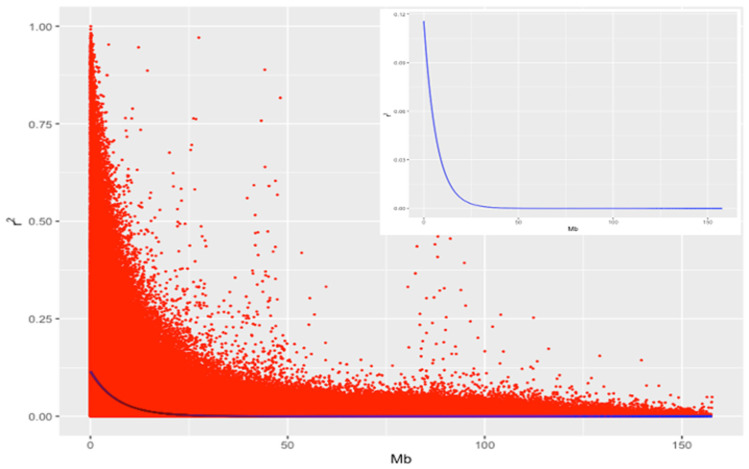
The linkage disequilibrium ($r^{2}$) among SNP markers plotted against the genetic distances (Mb) in Brangus heifers.

Pairwise, LD between 35,351 SNP markers were assessed using the squared correlation ($r^{2}$) between SNP markers. The average LD (SD) and genetic distance (SD) were 0.125 (0.156) and 0.503 (0.285) Mb within an interval of 1 Mb pairs across all chromosomes. The overall average for LD and genetic distances were 0.022 (0.054) and 29.060 (24.209) Mb, respectively. The distribution of LD ($r^{2}$) against the genetic distance (Mb) given in Figure 2 indicated a sharp decline with increases of the genetic distance between SNP. The association between the degree of decay in LD with the distance between SNP markers indicated a clear decreasing exponential trend with an increasing genetic distance (Figure 2). Higher LD values were obtained for SNP markers located in close proximity. For the SNP markers less than 0.1 Mb apart, the mean LD (SD) was 0.195 (0.224), and 11.22% of SNP marker pairs had an LD higher than 0.5. For the genetic distance between pairs of SNP markers at ranges from 0 to 0.1, 0 to 0.2, and 0 to 0.5 Mb, 11.22, 8.73, and 5.67% of SNP marker pairs showed a higher LD than 0.5, respectively.

McKay et al. [39] and El Hou et al. [36] reported that most of the studies based on bovine SNP data have shown that the average LD was close to zero for distances between SNP greater than 500 kb. Lu et al. [38] reported rapidly decreasing LD from 0.29 to 0.23 to 0.19 in Angus, 0.22 to 0.16 to 0.12 in Charolais, and 0.21 to 0.15 to 0.11 in crossbred cattle for the distances from 0–30 kb to 30–70 kb, and then to 70–100 kb, respectively. El Hou et al. [36] also found that the average LD values between pairs of SNP markers ranged from 0.079 to 0.121 for Charolais, Limousine, and Blonde d’Aquitaine cattle, and the average LD changed from 0.5 to 0.1 at distances from smaller than 15 kb to greater than 120 kb. Singh et al. [37] also calculated the average LD of 0.43 for the distance of less than 10 kb, and then it decreased to 0.21 for the distances of 25 to 50 kb for Vrindavani crossbred cattle.

### 3.2. Heritability Estimates from GBLUP, and the Bayesian (BayesA, BayesB, BayesC and Lasso) Methods in K-Means and Random Training Datasets

**Figure 3:**
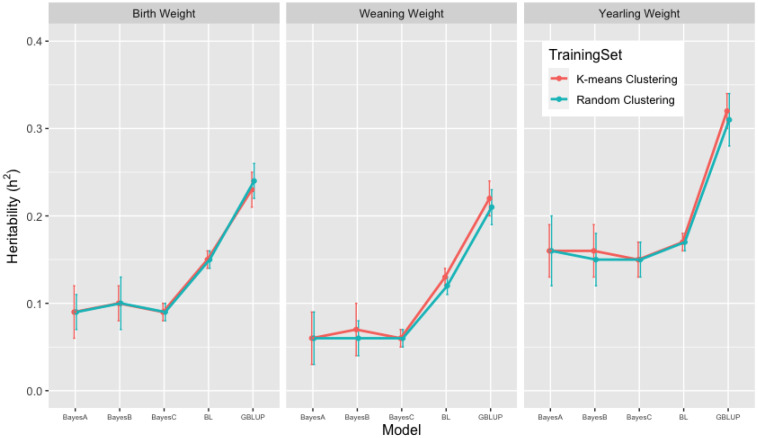
Mean estimates of heritability of birth weight (BW), weaning weight (WW) and yearling weight (YW) for growth traits from 10-fold k-means and random cluster training datasets using Genomic Best Linear Unbiased Prediction (GBLUP) and the Bayesian (BayesA, BayesB, BayesC, and Lasso (BL)) methods.

Heritability estimates of growth (BW, WW, and YW) and carcass (FAT, IMF, and LMA) traits from 10-fold k-means and random cluster training datasets were obtained from the GBLUP and the Bayesian (BayesA, BayesB, BayesC, and Lasso) methods. In the analyses of growth traits, the estimates of heritabilities across 10-fold k-means (random) cluster training datasets ranged between 0.20 (0.20) to 0.26 (0.30) from the GBLUP and between 0.05 (0.04) to 0.16 (0.16) from the Bayesian methods for BW; between 0.19 (0.17) to 0.24 (0.24) from the GBLUP; between 0.02 (0.02) to 0.13 (0.13) from the Bayesian methods for WW; between 0.30 (0.31) to 0.36 (0.36) from the GBLUP; and between 0.09 (0.08) to 0.22 (0.29) from the Bayesian methods for YW. In the analyses of carcass traits, the estimates of heritabilities across 10-fold k-means (random) cluster training datasets ranged between 0.28 (0.28) to 0.34 (0.33) from the GBLUP and between 0.09 (0.07) to 0.19 (0.19) from the Bayesian methods for FAT; between 0.31 (0.30) to 0.37 (0.37) from the GBLUP, and between 0.15 (0.14) to 0.25 (0.26) from the Bayesian methods for IMF; and between 0.30 (0.32) to 0.38 (0.42) from the GBLUP, and between 0.11 (0.11) to 0.23 (0.29) from the Bayesian methods for LMA (Table 2). Overall mean (±standard deviation) estimates of heritabilities for the growth traits for 10-fold k-means (random) cluster training datasets in Figure 3 were 0.23 ± 0.02 (0.24 ± 0.02) from the GBLUP and 0.09 ± 0.03 (0.09 ± 0.02), 0.10 ± 0.02 (0.10 ± 0.03), 0.09 ± 0.01 (0.09 ± 0.01) and 0.15 ± 0.01 (0.15 ± 0.01) from BayesA, BayesB, BayesC, and BL in the Bayesian methods for BW; 0.22 ± 0.02 (0.21 ± 0.02) and 0.06 ± 0.03 (0.06 ± 0.03), 0.07 ± 0.03 (0.06 ± 0.02), 0.06 ± 0.01 (0.06 ± 0.01), 0.13 ± 0.01 (0.12 ± 0.01) for WW; 0.32 ± 0.02 (0.31 ± 0.03) and 0.16 ± 0.03 (0.16 ± 0.04), 0.16 ± 0.03 (0.15 ± 0.03), 0.15 ± 0.02 (0.15 ± 0.02), 0.17 ± 0.01 (0.17 ± 0.01) for YW, respectively.

**Figure 4:**
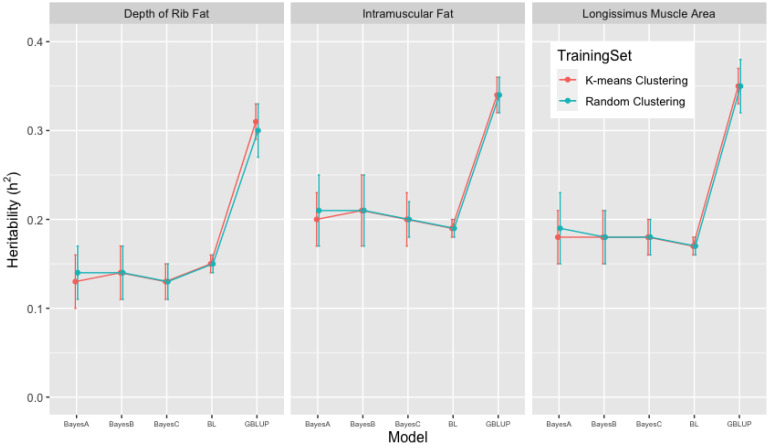
Means of heritability ($h^{2}$) estimates of depth of rib fat (FAT), intramuscular fat (IMF), and longissimus muscle area (LMA) for carcass traits from 10-fold k-means and random cluster training datasets using Genomic Best Linear Unbiased Prediction (GBLUP) and the Bayesian (BayesA, BayesB, BayesC, and Lasso (BL)) methods.

For the carcass traits, overall mean (±standard deviation) estimates of heritabilities in Figure 4 were 0.31 ± 0.02 (0.30 ± 0.03) from GBLUP and 0.13 ± 0.03 (0.13 ± 0.03), 0.14 ± 0.03 (0.14 ± 0.03), 0.13 ± 0.02 (0.13 ± 0.02) and 0.15 ± 0.01 (0.15 ± 0.01) from BayesA, BayesB, BayesC, and BL in the Bayesian methods for FAT; 0.34 ± 0.02 (0.34 ± 0.02) and 0.20 ± 0.03 (0.21 ± 0.04), 0.21 ± 0.04 (0.21 ± 0.04), 0.20 ± 0.03 (0.20 ± 0.02) and (0.19 ± 0.01) (0.19 ± 0.01) for IMF; 0.35 ± 0.02 (0.35 ± 0.03) and 0.18 ± 0.03 (0.18 ± 0.04), 0.18 ± 0.03 (0.18 ± 0.03), 0.18 ± 0.02 (0.18 ± 0.02) and 0.17 ± 0.01 (0.17 ± 0.01) for LMA.

As presented in Figure 3 and Figure 4, 10-fold k-means and random cluster training datasets resulted in very similar heritability ($h^{2}$) estimates for growth and carcass traits. The comparison of methods suggested that the GBLUP methodology yielded almost double the heritability ($h^{2}$) estimates than the Bayesian (BayesA, BayesB, BayesC, and Lasso) methods for growth and carcass traits within 10-fold k-means and random cluster training datasets. Within the Bayesian (BayesA, BayesB, BayesC, and Lasso) methods, the BL method resulted in higher estimates of heritability ($h^{2}$) than BayesA, BayesB, and the BayesC methods for growth traits; however, heritability ($h^{2}$) estimates for carcass traits were similar across the Bayesian methods. Peters et al. [9] reported the pedigree and genome-based estimates of heritabilities for growth and carcass traits by conducting GWAS analyses using the BayesC method and the SNP markers for the Brangus cattle of this study.

Pedigree-based estimates of heritabilities were similar with those from GBLUP for growth traits, but were higher than those from the Bayesian (BayesA, BayesB, BayesC, and Lasso) methods for growth and carcass traits. Genome-based (BayesC) estimates of heritabilies for growth and carcass traits were lower than those from GBLUP, but were similar with those from the other the Bayesian methods. The heritability ($h^{2}$) estimates from THE GBLUP for growth and carcass traits were in the range of heritability ($h^{2}$) estimates reported in the literature [9,40,41,42,43,44], and they suggested that GBLUP resulted in more reasonable heritability estimates than the Bayesian (BayesA, BayesB, BayesC, and Lasso) methods in the analyses using smaller subsets of the data [42].

### 3.3. Comparison of Genome-Wide Prediction Ability

**Figure 5:**
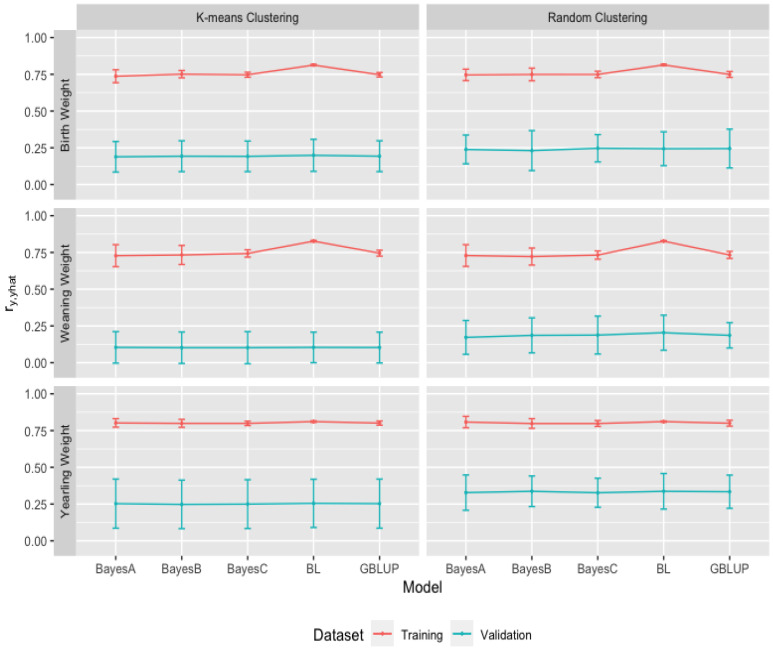
Predictive ability of Genomic Best Linear Unbiased Prediction (GBLUP) and the Bayesian (BayesA, BayesB, BayesC, and Lasso (BL)) methods for birth weight (BW), weaning weight (WW) and yearling weight (YW) for growth traits from 10-fold k-means and random cluster training datasets.

**Figure 6:**
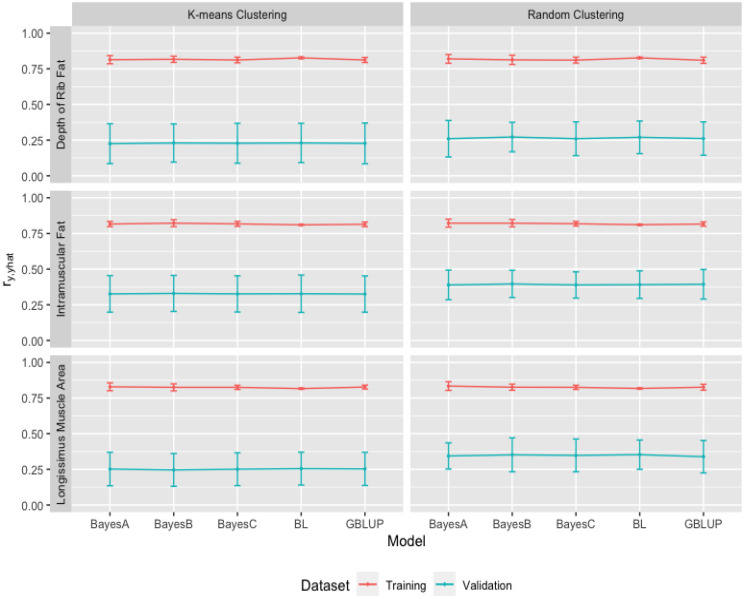
Predictive ability of Genomic Best Linear Unbiased Prediction (GBLUP) and the Bayesian (BayesA, BayesB, BayesC, and Lasso (BL)) methods for depth of rib fat (FAT), intramuscular fat (IMF), and longissimus muscle area (LMA) for carcass traits from 10-fold k-means and random cluster training datasets.

The GBLUP and the Bayesian methods were used to analyze growth (BW, WW, and YW) and carcass (FAT, IMF, and LMA) traits. The means and standard deviations of Pearson correlations ($r_{y,\hat{y}}$) between actual and predicted phenotypes for growth (BW, WW and YW) traits in Figure 5 and those for carcass (FAT, IMF, and LMA) traits in Figure 6 indicate the performance of genomic prediction from the GBLUP and the Bayesian methods when applied in the same 10-fold k-means and random cluster datasets for training and validation, respectively. As seen in Figure 5 and Figure 6, mean correlations for growth (BW, WW and YW) and carcass (FAT, IMF and LMA) traits from k-means and random cluster training data sets were similar across the GBLUP and the Bayesian methods. For growth (BW, WW and YW) traits, Figure 5 showed that BL method (on average, 0.813 ± 0.005, 0.827 ± 0.005, 0.811 ± 0.005 and 0.814 ± 0.005, 0.827 ± 0.004, 0.811 ± 0.004) resulted in higher Pearson’s correlations than GBLUP (on average, 0.747 ± 0.015, 0.745 ± 0.020, 0.801 ± 0.014 and 0.749 ± 0.020, 0.733 ± 0.024, 0.800 ± 0.020), BayesA (on average, 0.737 ± 0.044, 0.728 ± 0.075, 0.802 ± 0.029 and 0.746 ± 0.039, 0.729 ± 0.074, 0.808 ± 0.039), BayesB (on average, 0.751 ± 0.025, 0.733 ± 0.065, 0.799 ± 0.027 and 0.749 ± 0.043, 0.722 ± 0.058, 0.798 ± 0.033), and BayesC (on average, 0.747 ± 0.017, 0.743 ± 0.025, 0.799 ± 0.015 and 0.749 ± 0.022, 0.732 ± 0.028, 0.798 ± 0.020) methods within the 10-fold k-means and random cluster training datasets. For carcass (FAT, IMF and LMA) traits, Figure 6 showed that GBLUP (on average 0.812 ± 0.018, 0.814 ± 0.016, 0.827 ± 0.014 and 0.810 ± 0.022, 0.816 ± 0.015, 0.826 ± 0.021), BayesA (on average, 0.814 ± 0.029, 0.816 ± 0.018, 0.829 ± 0.028 and 0.820 ± 0.031, 0.822 ± 0.028, 0.834 ± 0.031), BayesB (on average, 0.817 ± 0.022, 0.822 ± 0.024, 0.825 ± 0.025 and 0.813 ± 0.033, 0.822 ± 0.025, 0.826 ± 0.022), BayesC (on average, 0.812 ± 0.020, 0.817 ± 0.017, 0.825 ± 0.015 and 0.811 ± 0.021, 0.818 ± 0.016, 0.825 ± 0.015), and BL (on average, 0.827 ± 0.006, 0.810 ± 0.003, 0.816 ± 0.004 and 0.827 ± 0.005, 0.811 ± 0.004, 0.817 ± 0.004) methods produced similar correlations ranging from 0.810 to 0.834 within the 10-fold k-means and random cluster training datasets; however, the correlations for carcass traits were higher than those for growth traits.

Predictive performance of the GBLUP and the Bayesian methods was explored by using the correlations from 10-fold k-means and random cluster cross-validation datasets. Figure 5 showed that GBLUP (on average, 0.193 ± 0.105, 0.103 ± 0.105, 0.253 ± 0.167 and 0.245 ± 0.132, 0.186 ± 0.086, 0.334 ± 0.113), BayesA (on average, 0.189 ± 0.104, 0.104 ± 0.107, 0.253 ± 0.167 and 0.239 ± 0.098, 0.172 ± 0.115, 0.328 ± 0.120), BayesB (on average, 0.193 ± 0.105, 0.102 ± 0.107, 0.248 ± 0.165 and 0.231 ± 0.136, 0.186 ± 0.119, 0.337 ± 0.104), BayesC (on average, 0.192 ± 0.104, 0.102 ± 0.109, 0.250 ± 0.166 and 0.247 ± 0.093, 0.188 ± 0.129, 0.327 ± 0.099), and BL (on average, 0.199 ± 0.109, 0.104 ± 0.104, 0.255 ± 0.164 and 0.244 ± 0.115, 0.204 ± 0.119, 0.337 ± 0.121) methods within the 10-fold k-means and random cluster cross-validation datasets resulted in similar correlations for growth (BW, WW and YW) traits. The ranges of the correlations within 10-fold k-means and random cluster cross-validations were from 0.189 ± 0.104 to 0.199 ± 0.109 and 0.231 ± 0.136 to 0.247 ± 0.093 for BW, 0.102 ± 0.107 to 0.104 ± 0.104 and 0.172 ± 0.115 to 0.204 ± 0.119 for WW, and 0.248 ± 0.165 to 0.255 ± 0.164 and 0.327 ± 0.099 to 0.337 ± 0.121 for YW. The ranges of correlations also indicated that the random cluster cross-validation resulted in a higher correlation than k-means cluster cross-validation, minimizing the genetic relationships among clusters. The trait of YW produced higher correlations than the traits of BW and WW within growth traits.

Figure 6 indicated that GBLUP (on average, 0.227 ± 0.143, 0.325 ± 0.127, 0.253 ± 0.116 and 0.261 ± 0.117, 0.394 ± 0.104, 0.339 ± 0.114), BayesA (on average, 0.225 ± 0.140, 0.326 ± 0.128, 0.252 ± 0.117 and 0.260 ± 0.128, 0.389 ± 0.104, 0.344 ± 0.092), BayesB (on average, 0.230 ± 0.134, 0.329 ± 0.126, 0.246 ± 0.115 and 0.272 ± 0.103, 0.396 ± 0.096, 0.352 ± 0.119), BayesC (on average, 0.228 ± 0.140, 0.326 ± 0.127, 0.251 ± 0.115 and 0.260 ± 0.118, 0.389 ± 0.092, 0.348 ± 0.115), and BL (on average, 0.230 ± 0.138, 0.327 ± 0.131, 0.255 ± 0.115 and 0.270 ± 0.114, 0.391 ± 0.097, 0.353 ± 0.103) methods within the 10-fold k-means and random cluster cross-validation datasets produced similar correlations for carcass (FAT, IMF and LMA) traits. The ranges of the correlations within 10-fold k-means and random cluster cross-validations were from 0.225 ± 0.140 to 0.230 ± 0.138 and 0.260 ± 0.128 to 0.272 ± 0.103 for FAT, 0.325 ± 0.127 to 0.329 ± 0.126 and 0.389 ± 0.104 to 0.396 ± 0.096 for IMF, and 0.246 ± 0.115 to 0.255 ± 0.115 and 0.339 ± 0.114 to 0.353 ± 0.103 for LMA. The ranges of correlations from the random cluster cross-validation were higher than those from the k-means cluster cross-validation. The trait of IMF produced higher correlations than the traits of FAT and LMA within carcass traits.

The predictive performances for growth (BW, WW, and YW) and carcass (FAT, IMF, and LMA) traits from k-means and random cluster training and validation datasets were found different within the GBLUP and the Bayesian methods, which depends on the genetic architecture of the traits. The similar predictive performances from the GBLUP and the Bayesian methods for growth (BW, WW and YW) and carcass (FAT, IMF and LMA) also suggested that the genetic structures of growth and carcass traits controlled by many genes with small effects. The carcass traits also resulted in the higher heritabilities and then higher predictive performances than growth traits. These results also revealed that the 10-fold k-means and random cluster cross-validation datasets resulted in significantly lower correlations than training datasets for growth (BW, WW and YW) and carcass (FAT, IMF and LMA) traits within the GBLUP and the Bayesian (BayesA, BayesB, BayesC, and Lasso) methods. The decrease in mean correlations for growth (BW, WW, and YW) and carcass (FAT, IMF, and LMA) traits ranged from 52% to 87% in the 10-fold k-means and random cluster cross-validation datasets. In addition, k-means cluster minimizing genetic relationship among cross-validation datasets produced lower correlations than the random cluster and the ranges of the decrease were from 16% to 22% for BW, 40% to 49% for WW, 23% to 26% for YW as growth traits, and 12% to 15% for FAT, 16% to 18% for IMF, and 25% to 30% for LMA as carcass traits across the GBLUP and the Bayesian methods.

**Figure 7:**
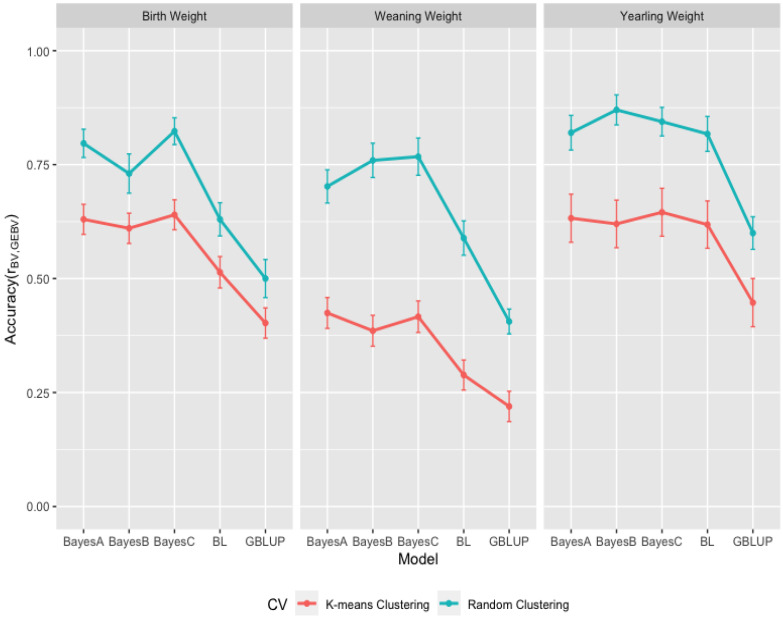
Accuracy of GEBV from Genomic Best Linear Unbiased Prediction (GBLUP) and the Bayesian (BayesA, BayesB, BayesC, and Lasso (BL)) methods for birth weight (BW), weaning weight (WW) and yearling weight (YW) for growth traits from 10-fold k-means and random cluster cross-validation datasets.

**Figure 8:**
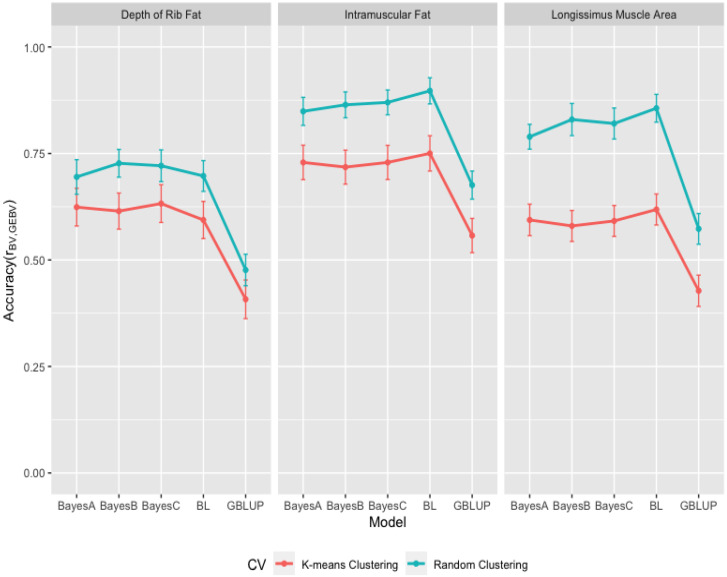
Accuracy of GEBV from Genomic Best Linear Unbiased Prediction (GBLUP) and the Bayesian (BayesA, BayesB, BayesC and Lasso (BL)) methods for depth of rib fat (FAT), intramuscular fat (IMF) and longissimus muscle area (LMA) for carcass traits from 10-fold k-means and random cluster cross-validation data sets.

The accuracies of GEBV from the GBLUP and the Bayesian methods were obtained from Pearson’s correlations ($r_{y,\hat{y}}$) between observed and predicted phenotypes divided by the square root of the estimated heritabilities and given in Figure 7 for growth and in Figure 8 for carcass traits within k-means and random cluster cross-validation datasets. As seen in Figure 7 for growth traits, the accuracies from the GBLUP and the Bayesian (BayesA, BayesB, BayesC, and Lasso) methods were 0.402 ± 0.033, 0.630 ± 0.033, 0.610 ± 0.033, 0.640 ± 0.033, 0.514 ± 0.034 within k-means clustering and 0.500 ± 0.042, 0.797 ± 0.031, 0.730 ± 0.043, 0.823 ± 0.029, 0.630 ± 0.036 within random clustering for BW; 0.220 ± 0.033, 0.425 ± 0.034, 0.386 ± 0.034, 0.416 ± 0.034, 0.288 ± 0.033 within k-means clustring and 0.406 ± 0.027, 0.702 ± 0.036, 0.386 ± 0.038, 0.768 ± 0.041, 0.589 ± 0.038 within random clustering for WW; and 0.447 ± 0.053, 0.633 ± 0.053, 0.620 ± 0.052, 0.645 ± 0.052, 0.618 ± 0.052 within k-means clustring and 0.600 ± 0.036, 0.820 ± 0.038, 0.870 ± 0.033, 0.844 ± 0.031, 0.817 ± 0.038 within random clustring for YW. As seen in Figure 8 for carcass traits, the accuracies from the GBLUP and the Bayesian (BayesA, BayesB, BayesC and Lasso) methods were 0.408 ± 0.045, 0.624 ± 0.044, 0.615 ± 0.042, 0.632 ± 0.044, 0.594 ± 0.044 within k-means clustering and 0.477 ± 0.037, 0.695 ± 0.040, 0.727 ± 0.033, 0.721 ± 0.037, 0.697 ± 0.036 within random clustering for FAT; 0.557 ± 0.040, 0.729 ± 0.040, 0.718 ± 0.040, 0.729 ± 0.040, 0.750 ± 0.041 within k-means clustering and 0.676 ± 0.033, 0.849 ± 0.033, 0.864 ± 0.030, 0.870 ± 0.029, 0.897 ± 0.031 within random clustering for IMF; and 0.428 ± 0.037, 0.594 ± 0.037, 0.580 ± 0.036, 0.592 ± 0.036, 0.618 ± 0.036 within k-means clustering and 0.573 ± 0.036, 0.789 ± 0.029, 0.830 ± 0.038, 0.820 ± 0.036, 0.856 ± 0.033 within random clustering for LMA.

The averaged accuracies of GEBV over all methods were 0.559 (0.696) for BW, 0.347 (0.645) for WW, 0.593 (0.790) for YW, 0.575 (0.663) for FAT, 0.697 (0.831) for IMF, and 0.562 (0.774) for LMA in k-means (random) cluster cross-validation datasets. As seen in Figure 7 and Figure 8, the random clustering approach resulted in higher accuracies of GEBV (24% for BW, 87% for WW, 33% for YW, 15% for FAT, 19% for IMF, and 37% for LMA) than the k-means clustering approach because of the higher relationship between training and validation datasets in random clustering. Habier et al. [45] executed the genome-wise analysis of milk yield, fat yield, protein yield, and somatic cell score, and indicated that the accuracy of GEBV decreased by reducing the genomic relationship between animals for the training and validation datasets. Saatchi et al. [19] found the accuracies of 0.554 and 0.700 for BW, 0.333 and 0.534 for WW, 0.356 and 0.573 for YW, 0.603 and 0.793 for FAT, 0.690 and 0.817 for Marbling, and 0.601 and 0.694 for LMA in the k-means and random cross-validation datasets from Angus cattle, and they suggested that minimizing the genetic relationships between animals from training and validation sets using k-means clustering resulted in the conservative accuracies of GEBV.

Daetwyler et al. [46] determined that the high accuracy of GEBV resulted from the family relationships rather than LD between SNP and QTL in a multiple-breed sheep population. Chen et al. [47] showed that individuals with close relatives in the training population had a higher accuracy of GEBV. Kang et al. [48] also reported the decreasing accuracy of GEBV with an increasing generation gap between the training and validation datasets. Zhou et al. [49] studied the factors affecting GEBV accuracy and reported that the genetic relationship between animals from cross-validation datasets created a more important effect than the LD between SNP and QTL on the accuracy of GEBV because the decrease in the accuracy of GEBV happened even when the LD between SNP increased.

The accuracies of GEBV from the GBLUP and the Bayesian (BayesA, BayesB, BayesC, and Lasso) methods averaged over all k-means and random clustering were 0.451, 0.713, 0.670, 0.732, 0.572 for BW, 0.313, 0.563, 0.572, 0.592, 0.439 for WW, 0.524, 0.726, 0.745, 0.745, 0.718 for YW in the growth traits, 0.442, 0.659, 0.671, 0.677, 0.645 for FAT, 0.617, 0.789, 0.791, 0.799, 0.824 for IMF, and 0.500, 0.692, 0.705, 0.706, 0.737 for LMA in the carcass traits. As presented in Figure 7 and Figure 8, the averaged accuracies of GEBV suggested that GBLUP resulted in lower accuracies of GEBV than the Bayesian methods within growth and carcass traits. The Bayesian methods exhibited quite similar accuracies of GEBV and the BayesC method for growth traits, and the Bayesian LASSO method for carcass traits provided higher accuracies of GEBV than other methods within the the Bayesian methods, respectively. Sun et al. [50] compared the GBLUP and the Bayesian methods using simulated data and found that the GBLUP had lower accuracy than BayesB and BayesCπ, and the Bayesian methods resulted in quite similar accuracies. Gao et al. [51] also reported that the Bayesian methods performed better accuracy of GEBV than the GBLUP methods in the genome analysis of milk production traits of Nordic Holstein cows. However, Chen et al. [52] reported that GBLUP performed better than Bayes B in the genomic analysis of carcass traits from Angus and Charolais beef cattle. Hayes et al. [53] also found that the GBLUP and the BayesB methods resulted in similar accuracies in a multibreed dairy population. Additionally, Ostersen et al. [54] reported no difference among the GBLUP, the Bayesian LASSO, and the Bayesian mixture methods based on 60,000 SNP data, and Ge et al. [55] reported the similar predictive accuracy for the GBLUP and the Bayesian methods for growth traits at weaning and yearling ages in Yaks. Although the predictive abilities of the GBLUP and the Bayesian methods were quite similar for growth and carcass traits and k-means and random clusters (Figure 5 and Figure 6) in the current study, the realized accuracies of the GEBV of the GBLUP and the Bayesian methods were not similar (Figure 7 and Figure 8) because of the heritability estimates from the GBLUP and the Bayesian (BayesA, BayesB, BayesC and Lasso) methods. As described by Rolf et al. [42] for the smaller subsets of the data used in analyses, robust and reasonable heritability estimates can be obtained from GBLUP methodologies compared to the Bayesian methods. The accuracies of GEBV from the GBLUP in this study were then found in the range of the theoretical predicted accuracies between 0.26 and 0.34 based on the heritability estimates of traits from 0.25 to 0.40 [56].

## 4. Conclusions

In order to explore the translation of genomic prediction for growth and carcass traits in Brangus cattle, the GBLUP and the Bayesian (BayesA, BayesB, BayesC and Lasso) methods were used to estimate GEBV for growth (BW, WW and YW) and carcass (FAT, IMF and LMA) traits within k-means and random clusters in this study. The heritability estimates from k-means and random cluster were quite similar across genomic prediction methods. The Bayesian methods underestimated the heritabilities between 0.06 and 0.21; however, the heritability estimates from GBLUP were between 0.21 and 0.35 for growth and carcass traits, and they parallel these types of estimates in the literature. Including low-density SNP markers with low minor allele frequency would cause a poor performance to estimate the heritabilities of traits with the Bayesian methods compared with GBLUP using genomic relationship. K-means cluster appears to minimize the genetic relationships among cross-validation datasets and yields lower correlations than in a random cluster. These results of the current study suggested that the level of genetic relationship between the training and validation data influences the prediction ability of genomic selection methods and the accuracy of GEBV. The prediction ability of the GBLUP and the Bayesian methods within k-means and random clusters were quite similar for growth and carcass traits; however, the Bayesian methods overestimated the accuracies of GEBV because of the lower estimates of the heritability of growth and carcass traits. However, the GBLUP resulted in more reasonable accuracy of GEBV for growth and carcass traits collected from Brangus heifers.

## Figures and Tables

**Table 1:** Descriptive statistics for growth and carcass traits in Brangus heifers.

Trait	Mean ± SE *	Minimum	Maximum
Birth weight (BW), kg	34.48 ± 0.19	18.03	50.94
Weaning weight (WW), kg	377.98 ± 1.81	201.37	549.56
Yearling weight (YW), kg	540.67 ± 3.45	225.51	769.67
Depth of rib fat (FAT), cm	0.57 ± 0.01	0.02	1.40
Intramuscular fat (IMF), %	4.81 ± 0.04	2.02	9.77
Longissimus muscle area (LMA), cm^2^	62.31 ± 0.41	27.22	91.19

* SE: Standard error of mean.

**Table 2:** Mean [minimum, maximum] estimates of heritability of birth weight (BW), weaning weight (WW), and yearling weight (YW) for growth traits, rib fat (FAT), intramuscular fat (IMF), and longissimus muscle area (LMA) for carcass traits from 10-fold k-means and random cluster training datasets across replications, using Genomic Best Linear Unbiased Prediction (GBLUP) and Bayesian (BayesA, BayesB, BayesC, and Lasso (BL)) methods.

	K-Means Cluster				
	THE GBLUP	BayesA	BayesB	BayesC	BL
Growth Traits					
BW	0.23 [0.20, 0.26]	0.09 [0.05, 0.13]	0.10 [0.07, 0.13]	0.09 [0.06, 0.11]	0.15 [0.14, 0.16]
WW	0.22 [0.19, 0.24]	0.06 [0.02, 0.11]	0.07 [0.02, 0.11]	0.06 [0.05, 0.08]	0.13 [0.12, 0.13]
YW	0.32 [0.30, 0.36]	0.16 [0.11, 0.22]	0.16 [0.09, 0.20]	0.15 [0.11, 0.19]	0.17 [0.15, 0.18]
Carcass Traits					
FAT	0.31 [0.28, 0.34]	0.13 [0.09, 0.19]	0.14 [0.09, 0.17]	0.13 [0.10, 0.17]	0.15 [0.14, 0.16]
IMF	0.34 [0.31, 0.37]	0.20 [0.16, 0.24]	0.21 [0.15, 0.25]	0.20 [0.16, 0.23]	0.19 [0.18, 0.20]
LMA	0.35 [0.30, 0.38]	0.18 [0.13, 0.23]	0.18 [0.11, 0.21]	0.18 [0.12, 0.21]	0.17 [0.15, 0.18]
	Random Cluster				
	GBLUP	BayesA	BayesB	BayesC	BL
Growth Traits					
BW	0.24 [0.20, 0.30]	0.09 [0.04, 0.15]	0.10 [0.04, 0.20]	0.09 [0.06, 0.13]	0.15 [0.14, 0.16]
WW	0.21 [0.17, 0.24]	0.06 [0.02, 0.17]	0.06 [0.02, 0.11]	0.06 [0.04, 0.09]	0.12 [0.11, 0.13]
YW	0.31 [0.26, 0.38]	0.16 [0.08, 0.29]	0.15 [0.08, 0.25]	0.15 [0.11, 0.21]	0.17 [0.15, 0.18]
Carcass Traits					
FAT	0.30 [0.23, 0.35]	0.14 [0.07, 0.23]	0.14 [0.06, 0.23]	0.13 [0.09, 0.18]	0.15 [0.14, 0.16]
IMF	0.34 [0.29, 0.39]	0.21 [0.13, 0.28]	0.21 [0.13, 0.31]	0.20 [0.15, 0.25]	0.19 [0.17, 0.20]
LMA	0.35 [0.29, 0.42]	0.19 [0.11, 0.29]	0.18 [0.11, 0.28]	0.18 [0.13, 0.21]	0.17 [0.15, 0.18]

## Data Availability

The data that support the findings of this study are available from the corresponding author, [S.O.P.], upon reasonable request.
